# T Cells in Atherosclerosis in Ldlr−/− and Apoe−/− Mice

**DOI:** 10.29245/2578-3009/2018/3.1144

**Published:** 2018-06-27

**Authors:** Godfrey S. Getz, Catherine A. Reardon

**Affiliations:** 1 Department of Pathology, The University of Chicago, Chicago, IL 60637, USA; 2 Ben May Institute for Cancer Research, The University of Chicago, Chicago, IL 60637, USA

## Abstract

Atherosclerosis is the underlying basis for most cardiovascular diseases. It is a chronic inflammation affecting the arterial intima and is promoted by hypercholesterolemia. Cells of both the innate and adaptive immune systems contribute to this inflammation with macrophages and T cells being the most abundant immune cells in the atherosclerotic plaques. In this review, we discuss the studies that examined the role of T cells and T cell subsets in *Apoe−/−* and *Ldlr−/−* murine models of atherosclerosis. While there is a general consensus that Th1 cells are pro-atherogenic and regulatory T cells are atheroprotective, the role of other subsets is more ambiguous. In addition, the results in the two models of atherosclerosis do not always yield similar results. Additional studies in the two murine models using cell specific gene manipulations are needed.

## Introduction

Atherosclerosis is a chronic inflammation primarily affecting the arterial intima and promoted by hypercholesterolemia, especially in mice. Both the innate and adaptive immune systems contribute to this inflammation. Initial events in atherogenesis involve the influx of monocytes/macrophages that take up modified apoprotein B containing lipoproteins to form foam cells and T cells into the subendothelial space of the aortic wall. As the plaque progresses, other immune cells also infiltrate the intima of the atherosclerotic plaque including dendritic cells, mast cells, NK cells and other minor immune cell types. The adventitia that surrounds the artery wall, particularly over arterial segments containing atherosclerosis, also contains immune cells, especially T and B cells. This brief review will focus on the adaptive immune system and the T cells in particular. Robust atherosclerosis can occur in both LDL receptor deficient (*Ldlr−/−*) and apoprotein E deficient (*Apoe−/−*) murine models of atherosclerosis in the absence of the cells of the adaptive immune system^1–5^. The fact that atherosclerosis readily occurs in the absence of T and B lymphocytes is probably attributable, at least in part, to the fact that in immune competent animals both pro-inflammatory and anti-inflammatory T cells are active and effect some balance between these two cell types. Whether this balance is biased toward one or other of these cells will determine the atherosclerosis outcome. In different experimental contexts and arterial sites, this balance may vary.

Until recently^6^ it has been difficult to induce significant atherosclerosis in wild type mice. Thus, the study of experimental atherosclerosis, especially the full spectrum of atherosclerosis, has been mostly undertaken with either *Apoe−/−* or *Ldlr−/−* mice in the C57BL/6 genetic background. The LDLR is a ubiquitously expressed cell-surface receptor that recognizes apoB100 and apoE on lipoprotein particles and mediates their clearance from the plasma. A deficiency in either the receptor or ligand facilitates the induction of hypercholesterolemia, which is a major factor that drives atherogenesis. *Apoe−/−* mice are hypercholesterolemic and develop atherosclerosis while maintained on a standard chow diet, though both are accentuated by feeding a high fat, high cholesterol diet, the so called Western type diet (WTD). In the *Ldlr−/−* model significant hypercholesterolemia and atherosclerosis occurs only when fed the WTD. In *Apoe−/−* mice the most prominent lipoprotein is the large VLDL remnant, which is rich in cholesteryl ester and apoB48. On the other hand, in the *Ldlr−/−* mouse, much of the cholesterol is carried by LDL, a smaller lipoprotein containing apoB100 as the dominant apoprotein. In an attempt to explore the role of lipoprotein size in atherogenesis, Steve Young and colleagues developed murine models expressing only apoB100 in both the *Apoe−/−* and *Ldlr−/−* background^7^. While both strains had almost identical total serum cholesterol levels, the cholesterol in the *Apoe−/−apoB*^*100/100*^ mice was carried in a smaller number of large lipoproteins and in the *Ldlr−/−apoB*^*100/100*^ mice in a larger number of smaller lipoprotein particles. Atherosclerosis was more extensive in the *Ldlr−/−apoB*^*100/100*^ model suggesting that the more permeable lipoprotein (i.e., LDL) more readily generates lesions. Of the two models *Apoe−/−* mice have been more frequently used for studies of the inflammatory component of atherosclerosis. For example, in a recent review by Klaus Ley and colleagues^8^, approximately twice as many studies of *Apoe−/−* mice were referenced than *Ldlr−/−* mice.

## T cells and T cell subclasses in atherosclerosis

In wild type mice, there are virtually no T cells in the intima. What T cells are found in the normal artery wall in the absence of lesions are mostly in the adventitia surrounding the artery^9^. When CFSE-labeled splenocytes are adoptively transferred into mice with atherosclerosis they first appear in the adventitia^9^. Ultimately, they represent the second largest leukocyte population in the aortic wall after macrophages. L-selectin and the chemokine/chemokine receptor molecules involved in T cell migration into the arterial wall are CCL5/CCR1-CCR5, CCL19-CCL20/CCR7, CXCL10-CXCR3, and CXCL16/CXCR6^7^. Genetic deletion of the ligand or receptor or interference with their interaction has been shown in many, but not all, studies to reduce T cell influx into the aorta and reduce atherosclerosis. However, it should be noted that these studies did not involve cell-specific deletion of the ligand/receptor and many of these proteins are expressed on cells other than T cells.

There are several subsets of T cells that express inflammatory or anti-inflammatory mediators. The most clearly defined proinflammatory T cell is the IFNγ, TNFα, IL-12 and IL-18 producing T helper cell 1 (Th1). These cells are the most prevalent T cell subtype in the atherosclerotic intima, where by virtue of their cytokine production they influence the other cells of the evolving atheroma and enhance lesion development^10–13^ Table 1. The T cells that accumulate in the lesions are reactive to atherosclerosis related antigens, such as oxidized LDL (OxLDL), apoB100, and HSP60/65^14–16^. Indeed, the adoptive transfer of splenic CD4^+^ T cells from atherosclerotic mice or wild type mice immunized with OxLDL into immune deficient *Apoe−/−* mice exacerbates atherogenesis to a greater extent than do T cells from normal mice^17,18^. Additional T cell subtypes that express the αβTCR have also been examined for their role in atherosclerosis, based primarily by manipulation of the level or signaling by the cytokines they produce. This includes Th2 cells that produce IL-4, IL-5, and IL-13 and Th17 cells that produce IL-17A, IL-17F and IL-22 among other cytokines. A minor T cell subset expressing the γδ TCR (γδ T cells) have also been examined. The study of the impact of each of these T cell subtypes on atherogenesis yields ambiguous results; most of the Th2 cytokines are pro-atherogenic, but some are atheroprotective (e.g., IL-5)^19^ and Th17 cells have demonstrated both pro-atherogenic and atheroprotective effects^20^. The basis for these different results may depend upon the precise experimental contexts in which they are studied, including maturation level of the lesion and the arterial site. For example, aortic γδT cells in *Apoe−/−* mice subject to WTD for 10 weeks were increased in the aorta compared to chow fed mice. However, the absence of these cells in *Apoe−/−δT−/−* mice had no effect on atherosclerosis^21^. Another study^22^ also showed the predominance of these cells in early lesions of the *Apoe−/−* mice. In this latter study, a reduction of early lesion growth in the aortic root and aortic arch was observed in the *Apoe−/−δT−/−* mice. All of the T cells discussed above are CD4^+^ cells. CD8^+^ T cells have also been detected in atherosclerotic lesions, but at lower levels than CD4^+^ T cells. While there is no clear consensus on their effect on the size of atherosclerotic lesions^23^, they may play a role in plaque destabilization in more advanced lesion, perhaps due to the secretion of granzyme and/or perforin^24^.

There are several potential issues that may contribute to the lack of consensus on the role of the less prevalent T cell subtypes on atherosclerosis. First, it is worth noting that lesions evolve at different rates at different vascular sites, with atherosclerotic lesions in the aortic root being the first to appear in mice^25^. As atherogenesis progresses, lesions appear in the aortic arch, the innominate artery, and the carotid arteries. By the time the abdominal aorta develops lesions, essentially the whole aorta may be involved. Thus, the maturity of the lesion at the site of atherosclerosis examined may vary in the different studies. It should be noted that coronary artery lesions, which readily form in humans, are not characteristically found in the *Apoe−/−* or *Ldlr−/−* mice. Second, the immune cell populations in the lesions are dynamic. For example, an early study by Roselaar and Daugherty^26^ enumerated lymphocyte numbers in the proximal aorta of *Apoe−/−* and *Ldlr−/−* mice at various times after initiation of a high cholesterol diet containing cholic acid. Even though the total plasma cholesterol was twice as high in *Apoe−/−* mice than in *Ldlr−/−* mice, the arterial wall T cell density was lower in the *Apoe−/−* model. Further they observed that T cell density decreased over time and that the decline was much more rapid in *Apoe−/−* mice than in the *Ldlr−/−* model. In this study, T cell subsets were not enumerated. The dynamic nature of the T cell complement during atherogenesis makes it more difficult to assess the functional contribution of T cells or their subtypes at each stage of atherogenesis. Such a limitation needs to be borne in mind in assessing the role of the adaptive immune system during the evolution of atherogenesis in each model. This study also points to possible inherent differences in the immune cells in the *Apoe−/−* and *Ldlr−/−* mice. Finally, the possible plasticity of T cell subsets overtime in atherosclerotic lesions could complicate this analysis^27^. Unfortunately, no comprehensive studies of T cells and T cell subsets have yet been undertaken under comparable experimental conditions, such for example as was done using the models described by Young and colleagues^7^. In any event this is not an easy comparison to accomplish.

The CD4^+^ regulatory T (Treg) cells, which produce the anti-inflammatory cytokines IL-10, TGFβ, and IL-35, are generally recognized by their expression of the canonical transcription factor FoxP3. The major Treg subsets are natural Treg (nTreg) and induced Treg (iTreg) cells that are induced in the context of inflammation. These cells suppress the proliferation and activity of effector T cells and the function of antigen presenting cells, especially dendritic cells. Numerous studies have demonstrated a role of Treg cells in protecting against atherosclerosis and they are being discussed as possible therapeutic targets for reduction of atherosclerosis^28–30^. Recently Treg cells that recognize a specific apoB100 peptide presented in MHC-class II molecules have been detected in both humans and *Apoe−/−* mice^31^. However, FoxP3 expression does not always reflect a suppressor cell. A recent report indicates that CD4^+^ T cells with the phenotype CCR5^+^CD25^−^Tbet^+^FoxP3^lo^ do not have properties of suppressor cells and adoptive transfer of these effector cells into *Apoe−/−* mice exacerbates rather than suppresses lesion growth^32^. Two very recent publications have highlighted the plasticity of Treg cells. Atherogenic dyslipidemia may promote autoimmune T follicular helper (Tfh) cell formation. Transplantation of bone marrow from lupus prone mice into *Apoe−/−* or *Ldlr−/−* recipient mice increases the level of plasma IgG2c antibodies and CXCR3^+^ Tfh cells^33^. The differentiation of these cells is apparently dependent on the production of IL-27 by dendritic cells. The laboratory of Catherine Hedrick has demonstrated the potential of Treg cells to be converted into Tfh cells during atherogenesis^34^. Using a Treg lineage tracker model in the *Apoe−/−* background, these authors showed that a significant number of Treg cells lose their expression of Foxp3 and are converted into Tfh cells with a CXCR5^+^PD1^+^BCL6^+^CD44^hi^CD62L^lo^CD4^+^ phenotype during lesion development. The formation of these cells is reduced by blocking ICOS signaling with an antibody to the ligand and mice deficient in Tfh cells have reduced atherosclerosis indicating that Tfh cells are proatherogenic. These cells have also been implicated in the formation tertiary lymphoid organs in the aortic adventitia^35^ (see below). Interestingly the treatment of atherogenic mice with lipid free apoA-I reduces the conversion of Treg cells to Tfh cells, mostly by reducing their cholesterol content and impacting membrane microdomains. This changes the homeostasis of IL-2/IL2R signaling that is important for the maintenance of Treg cells. The impact of apoA-I on the Treg cells is not dependent on changes in plasma HDL levels.

## T cell costimulatory/coinhibitory molecules

When T cells are activated, not only is the T cell receptor engaged, but also costimulatory or coinhibitory molecules modulate the response^36,37^. Most studies involving genetic deletion or inhibition of the interaction between the molecular pairs in murine atherogenic models have shown that the costimulatory molecules OX40-OX40L, CD137-CD137L, CD28-CD80/CD86 and CD40-CD40L are proatherogenic and the coinhibitory molecules CTLA4-CD80/CD86, CD27-CD70, and PD-1-PDL1/2 are atheroprotective. The CD40-CD40L costimulatory molecules have been extensively studied. CD40L is elevated in activated T cells and initial studies were based upon the perception that these costimulatory molecules reflected T cell involvement. Interruption of signaling through these molecules, mostly with anti-CD40 ligand antibody, almost uniformly results in the formation of a stable lesion phenotype (increased collagen, vascular smooth muscle cells and fibrous cap), regardless of the mouse model used or the duration of treatment^38^. This is a beneficial effect since unstable lesions are prone to rupture, resulting in clinical events such as myocardial infarctions and stroke. In the *Ldlr−/−* model, early and mature lesions were reduced in size by anti-CD40L treatment indicating their involvement in both lesion initiation and progression. On the other hand, in the *Apoe−/−* model antibody treatment had no effect on early or mature lesion size but the global knockout of the ligand reduced the size of mature but not early atherosclerotic lesions. With the global knockout of CD40 in the *Ldlr−/−* background, there was no protection against lesion formation, while atheroprotection was observed in the *Apoe−/−* background. The reconciliation of these apparently complex results probably lies with the fact that both CD40 and CD40L are expressed in cells other than T cells, including platelets, B cells, monocytes, macrophages, smooth muscle cells and endothelial cells, cells relevant to atherogenesis, as well as the observation that receptors other than CD40 recognize CD40L, including integrins. However, the basis for the difference between the *Ldlr−/−* and *Apoe−/−* models is not clear. It is only by manipulating CD40 and CD40L in a cell specific fashion that this complexity is likely to be resolved.

In addition to preventing the initiation and progression of atherosclerosis, identifying pathways that can be targeted to promote regression of atherosclerosis are of therapeutic interest. Interruption of signaling by the costimulatory molecules OX40-OX40L caused a regression of atherosclerosis when *Ldlr−/−* mice with established atherosclerosis were treated with anti-OX40L antibodies and returned to the low-fat chow diet to reduce hypercholesterolemia^39^. Interestingly this was accompanied by a reduction of adventitial T cells.

The ligands of the Lymphotoxin/LIGHT costimulatory family are expressed by T cells, as well as other immune cells, and this costimulatory family has also been implicated in atherosclerosis. There are two subtypes of the ligand lymphotoxin (LT); LTα_3_, a secreted homotrimer of lymphotoxin a (LTa) chains and LTα_2_β_1_ a membrane bound heterotrimer consisting of two LTα and one lymphotoxin β (LTβ) chain. LTα_3_ interacts with TNF receptors, while LTα_2_β_1_ mainly interacts with the lymphotoxin β receptor (LTβR), which is fairly widely expressed including on macrophages, epithelial and stromal cells, such as endothelial cells and smooth muscle cells. LIGHT, the other ligand in this family, interacts with LTβR and Herpes virus entry mediator (HVEM). In *Ldlr−/−* mice with global deficiency of LTα, thus, deficient in both lymphotoxins, or with T cell specific deficiency of LTβ a reduction in plasma lipid levels was observed after 12 weeks on WTD (unpublished observations). The similarity of lipid changes in global *LTα* deficiency and T cell specific *LTβ* deficiency suggests strongly that the T cell mediates these lipid responses. Atherosclerosis in these models yields quite complex results. In the global deficiency of *Lta* there is reduction in aortic root atherosclerotic lesions, which could very well be attributable to the plasma lipid reductions. However, this could not be the case for the T cell specific *Ltb* knockout, which exhibits an early elevation of lesion size in the aortic root despite the lower plasma lipids. In contrast to *Ldlr−/−* mice with deficiencies of the lymphotoxin ligands, WTD feeding of *Ltbr−/−Ldlr−/−* animals had no effect on plasma lipids or aortic root atherosclerosis.

In contrast to these results in *Ldlr−/−* mice, mice deficient in LTβR in the *Apoe−/−* background exhibit reduced aortic root and whole aortic atherosclerosis, with a reduction in the macrophage content of the lesions^40^. An increase in Ly6C’^lo^ monocytes in the blood was observed also, likely due to decreased influx of this monocyte subset into atherosclerotic lesions. The authors did not examine T cells in this study. The animals were fed a WTD for 15 weeks, a duration of feeding that produces very advanced lesions, more advanced than those observed in *Ldlr−/−* mice fed diet for 12 weeks. Interestingly, an increase in atherosclerosis associated with the deficiency of *LTβR* was observed in chow fed *Apoe−/−* mice, particularly in the descending and abdominal aorta of aged mice^41^.Thus, it appears that the effects of global LTβR deficiency on atherosclerosis phenotype in *Ldlr−/−* and *Apoe−/−* models differ from each other in complex ways. Understanding this complexity will require studying the selective deficiency of these ligands or receptors in a variety of cell types in both murine models of atherosclerosis.

LIGHT is another ligand in this costimulatory family. We have studied LIGHT expressed as a transgene in T cells and shown that it substantially down regulates the expression of hepatic lipase, a plasma enzyme involved in HDL metabolism^42^. We have also shown that the expression of LIGHT primarily by hepatocytes in mice using an adenovirus also reduces hepatic lipase expression independent of T cells^43^. In the presence of LIGHT overexpressing T cells in WTD fed *Ldlr−/−* mice a novel large HDL-like lipoprotein, HDL1, which is almost devoid of apoA-I, but is rich in apoE appears in the plasma. We postulate that hepatic lipase plays an important role in the metabolism and clearance of HDL1 particles. Indeed, in prior studies of hepatic lipase deficient mice in the *Ldlr−/−* background, a similar large HDL was noted^44^. T cell specific LIGHT transgenic *Ldlr−/−* animals exhibit a reduction of both aortic root and ascending aorta atherosclerosis with 6 weeks of WTD feeding. The in vivo interaction of LIGHT and HVEM is suggested by the lipoprotein profile of global knockouts of each of these genes in *Ldlr−/−* mice. In both cases, HDL levels decline significantly with 12 weeks, but not 6 weeks, of WTD feeding (unpublished data).

The aortic adventitia is being recognized as participating in the atherogenic process^45^. The long-term study of *Apoe−/−* mice reveals the appearance of adventitial tertiary lymphoid organs (ATLO), especially in the vicinity of atherosclerosis of the abdominal aorta^46,47^. Aortas of chow fed *Apoe−/−* mice were examined at 16, 32, 52 and 78 weeks of age. Atherosclerotic lesions in the abdominal aorta are very small in the 52 week old animals, but are very obvious at 78 weeks of age. ATLOs are not present in lesion free regions of the aorta and have a strong preference for the abdominal aorta at all stages of their development. The basis for this preference is not clear. The mature ATLO has lymphoid follicles with separate B and T cell regions, the latter being at the margins of the B cell cluster and the adventitia. As the ALTOs also contain macrophages, dendritic cells and B cells, these structures may provide a space in close proximity to the atherosclerotic lesion for the interaction of T cells with antigen presenting cells^45^. As the lesion advances, the T cell level in the atherosclerotic intima declines while that in the overlying adventitia increases, so that in advanced lesion areas the ratio of adventitial T cells/lesion T cells may be as high as 80:1. A significant percentage of the T cells in the ATLO are iTregs^46,47^. The development of the ATLO appears to depend on LTβR-mediated activation of medial smooth muscle cells to promote the expression of chemokines CCL21 and CXCL13 to recruit T and B cells^41,48^. How apoE deficiency impacts the development of these ATLOs is not clear. No such structures have been described in *Ldlr−/−* mice, though sufficiently long-term experiments may not have been conducted in this model.

Autoimmune skin lesions have been reported in high fat diet fed *Apoa1−/−Ldlr−/−* mice^49^. These mice develop a lymphadenopathy of the peripheral lymph nodes with increased levels of T and B cells, dendritic cells and macrophages. T cells and the other immune cells except macrophages are enriched in cholesteryl esters^50^. These changes are accompanied by a decrease in Treg cells in the peripheral lymph nodes. ApoA-I is the major apoprotein on HDL, a lipoprotein that, among other functions, promotes the efflux and transport of excess cellular cholesterol in peripheral cells to the liver. Treatment of the mice with apoA-I, reduces the cholesterol content of the lymph nodes and decreases total immune cell content with decreased effector T cells and increased Treg cells^50,51^. Our studies of *Apoal−/−Apoe−/−* double knockout animals do not replicate these skin lesions. This may indicate a differential response of *Ldlr−/−* and *Apoe−/−* mice to the absence of apoA-I. However other differences in experimental design, including the fat content of diet (10% palm oil - mixture saturated and unsaturated fatty acids vs. 42% milk fat- primarily saturated fatty acids) and length of time on diet (12 weeks vs 10 weeks) may also have contributed to these different results.

## Natural killer T (NKT) cells

NKT cells are another subset of T cells. They are a bridge between innate and adaptive immune systems and can produce both Th1 and Th2 cytokines upon activation. The T cell receptor on NKT cells is more restricted than is the case for conventional T cells. There are two major types of NKT cells. The invariant NKT cells (or iNKT cells) contain a semi-invariant T cell receptor that in mice contains the Vα14-Jα18 chain and a limited set of β-chains. These are distinguished from the minor set of variant NKT cells (vNKT cells), which have a more varied constellation of T cell receptors. These receptors recognize glycolipid and phospholipid antigens presented by the MHC class 1-like CD1d molecule on antigen presenting cells. The lipid antigens may be derived from microbes or from endogenous sources. The exogenous antigen that has been most widely studied experimentally is alpha galactosyl ceramide (αGalCer), derived from a marine sponge, which specifically activates iNKT cells. The antigens responsible for the activation of vNKT cells are less well characterized. NKT cells are found in many tissues with the cells being most prevalent in the liver, intestine, spleen, and adipose tissue. iNKT cells are heterogeneous as outlined in our recent review^52^. Two notable points. NKT1 (Tbet^+^) cells are the most prominent iNKT cell subtype in the C57BL/6 mouse strain, the strain used for most atherosclerosis experiments. Like other T cells, iNKT cells are dynamic, often declining with the progression of chronic inflammations such as atherosclerosis and obesity. Thus, the time of the analysis of the aorta may be an important determinant of the reported outcome of experiments on the impact of these cells on atherosclerosis.

Endogenous NKT cell antigens may be present on VLDL, which are taken up by cells via LDLR recognition of apoE on the lipoprotein^53^. Although this is not the sole route for the delivery of NKT cell antigens, the possible involvement of the apoE/LDLR pathway suggests that the activation of NKT cells may be suboptimal in mice lacking either the ligand or receptor. This notwithstanding, almost all murine experiments attempting to assess the role of these cells in atherogenesis have employed one of these two models. For the most part, murine studies have found that iNKT cells, whether activated by exogenous αGalCer, by overexpressing Vα14 by transgenesis, or by deficiency of these cells using *Ja18−/−* or *Cd1d−/−* mice, have pointed towards a proatherogenic role of iNKT cells. These results have been summarized in recent reviews^52,54^. However, there are some exceptions. For example, Aslanian and colleagues^55^ in experiments with *Cd1d−/− Ldlr−/−* mice found that early aortic root lesions were reduced 4 weeks after the initiation of the WTD, but not at later times of lesion progression. Using similar mice, we found the opposite results for early lesions in the aortic root, while ascending aorta lesions were reduced in both *Cd1d−/− Ldlr−/−* and *Ja18−/− Ldlr−/−* at 12 weeks of WTD feeding (unpublished data). Given that microbial antigens may activate NKT cells, it is not altogether surprising that contradictory results may be found in different vivaria. VanderLaan et al^56^ found that the adoptive transfer of splenocytes from Vα4 transgenic mice into *Ldlr−/− Rag1−/−* enhanced atherosclerosis in the aortic root, yet when the transgenic mice were crossed with *Ldlr−/−* mice, the increase in lesion area was seen in the innominate artery rather than the aortic root. LDL isolated from *Ldlr−/−* mice was shown to stimulate an NKT cell clone in culture, suggesting the presence of an NKT cell antigen in the circulating LDL.

One study observed an atheroprotective role of NKT cells but only in *Ldlr−/−* mice. In this study, the injection with αGalCer substantially reduced lesions induced in the carotid artery by collar placement in *Ldlr−/−* mice, while no effect was observed in *Apoe−/−* mice^57^. The explanation for this discordance between the two strains is not absolutely clear, though it has been suggested that the effect in *Ldlr−/−* mice may be due to the greater stimulation of the production of anti-inflammatory cytokines, while the unresponsiveness of *Apoe−/−* animals may be related to the anergic state of NKT cells in this model^58^.

Recent studies have pointed to the release of cytotoxic proteins, in particular granzyme and perforin, from NKT cells in promoting atherosclerosis. These proteins can induce apoptosis, which if unaccompanied by increased efferocytosis, would enhance lesion formation^59,60^.

In summary, as with T cell subsets, there remain many incompletely explained subtleties in the impact and mechanism of action of NKT cells on atherosclerosis. Understanding these subtleties will require further deep genetic and functional dissection

## Conclusion

Although there is little dispute about the proatherogenic role of CD4^+^ Th1 cells and for the most part of iNKT cells, there are many questions that remain. These questions include: how the cells influence atherosclerosis and how or if differences in the level or function of the T cell subtypes affect atherosclerosis at various stages of lesion development and in different regions of the aorta, particularly in view of the dynamic changes in T cells as the lesion progresses. The role of Th2 cells on the evolution of atherosclerosis is yet to be fully explained. Issues related to the interaction of proatherogenic T cells with other cells of both the innate and adaptive immune system also remain to be fully resolved. Resolution of these issues will likely require the development of new in vivo approaches, including experiments with cell specific gene manipulation. For example, overexpression of TGF in T cells or macrophages yield different outcomes^61,62^. Most important for the theme of this review is to differentiate between the two murine models of atherogenesis in relation to the inflammatory and anti-inflammatory role of various T cell subsets. Ideally these comparative studies should be performed in the same laboratory because of the possible involvement of the microbiome in the inflammatory process. As neither of the models reviewed here is ideal, many of these studies probably need to be carried out with another model, perhaps the apoB100 only mouse. Finally, in consideration of the relevance of these murine studies to human atherosclerosis, it is noteworthy that most murine studies address relatively early stages of atherogenesis, while human research on vessels mostly deals with well established lesions.

## Figures and Tables

**Table 1.** Effect of T cell subsets on atherosclerosis in murine atherosclerotic models.

T cell Subtype	Effect on Atherosclerosis
Th1	Pro-atherogenic Reactive to atherosclerosis relevant antigens
Th2	Considered pro-atherogenic based on role of cytokines produced. Exception is IL-5, which is atheroprotective
Th17	Pro-atherogenic and atheroprotective effects observed Effect may be context dependent
γδT cells	No effect or pro-atherogenic depending on arterial site
CD8+ T cells	No clear consensus May have role in lesion destabilization
Treg cells	FoxP3^hi^ cells are atheroprotective FoxP3^lo^ cells are pro-atherogenic
Tfh cells	Pro-atherogenic May be derived from FoxP3+ Treg cells during atherogenesis
iNKT cells	Pro-atherogenic in most studies Cytotoxic protein released from the cells may promote atherosclerosis
